# Rhabdomyosarcoma of the posterior chest wall in a newborn: a case report

**DOI:** 10.4076/1757-1626-2-6818

**Published:** 2009-07-02

**Authors:** Onkar Singh, Shilpi Singh Gupta, Vijay Upadhyaya, Shashi Shankar Sharma, Brijesh Kumar Lahoti, K Raj Mathur

**Affiliations:** 1 Department of Surgery, M.G.M Medical College & M.Y. HospitalIndore - 452001India; 2 Division of Paediatric Surgery, M.G.M Medical College & M.Y. HospitalIndore - 452001India

## Abstract

Rhabdomyosarcoma is the most common soft tissue malignancy of childhood, but may occur extremely rarely in the neonatal period. There are only a few reports of rhabdomyosarcoma in neonates. Although, it may arise anywhere in the body, the head and neck, and genitourinary regions are the most frequent sites. Truncal and chest wall rhabdomyosarcoma is relatively rare occurrence. We report a neonate with embryonal rhabdomyosarcoma arising from the posterior chest wall muscles at birth. Computer Tomography scan raised the possibility of rhabdomyosarcoma or neurofibroma, fine-needle aspiration cytology was inconclusive. Total excision was done and chemotherapy given. At 6 months child is without recurrence.

## Introduction

Rhabdomyosarcoma (RMS) is the most common soft tissue malignancy of childhood, but may occur extremely rarely in the neonatal period [1,2]. There are only a few reports of RMS in neonates [3,4]. Although, it may arise anywhere in the body, but it has a predilection for the head and neck area, genitourinary tract and the extremities [2]. Chest wall is a rare site for RMS [2,5]. We report a neonate with embryonal RMS arising from the posterior chest wall muscles at birth. CT scan raised the possibility of RMS or neurofibroma, FNAC was inconclusive. Total excision was done and chemotherapy given. At 6 months child is without recurrence. Case is reported because of extreme rarity of RMS to occur in neonates and that too in chest wall.

## Case presentation

**Figure 1.**
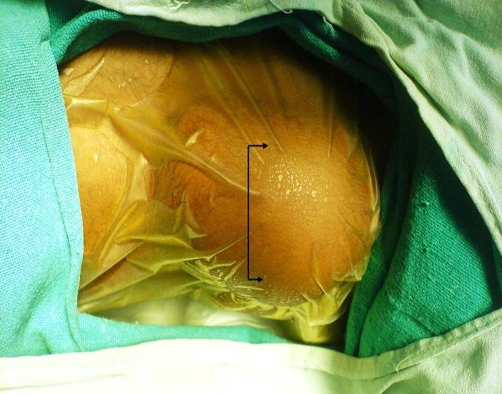
Pre-operative photograph of the back of a neonate showing a mass of size 6 × 5 cm situated on the posterior chest wall in right para-vertibral and right scapular region.

**Figure 2.**
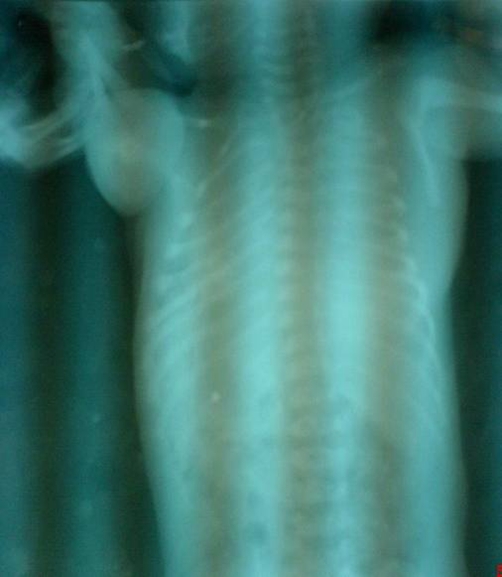
Babygram of the patient showing a soft tissue shadow on right scapular region.

**Figure 3.**
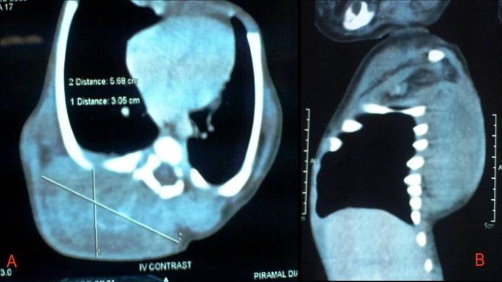
**(A)** & **(B)** (Transverse and Sagital views): CT-scan chest and upper abdomen of a newborn who presented with a hard mass on his back of chest, showing a minimally enhancing low-attenuating soft tissue mass, localized in posterior chest wall in right para-vertibral and right intra-scapular region.

**Figure 4.**
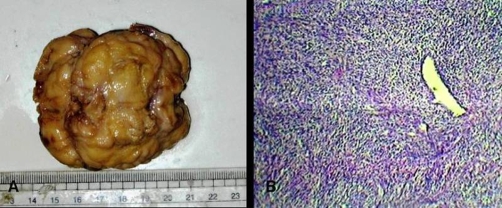
**(A)** Post-operative specimen after total excision of the mass. **(B)** Histopathological examination showing features of embryonal RMS.

An Indian, Hindu male baby was referred from the nursery, just three hours after birth with a congenital swelling on right side of his upper back. Baby was the first born child to a 29-year-old healthy mother at 38 weeks of gestation by normal vaginal delivery. On examination, there was a firm, oval, non-tender, slightly movable mass of size 6 × 5 cm situated on the posterior chest wall in right para-vertebral and right intra-scapular region (Figure 1). Lymph nodes in axillary or supra-clavicular region were not clinically palpable. It was not associated with any congenital anomaly. Chest X-ray showed an oval soft tissue shadow at the involved site (Figure 2). On ultrasound a homogenous soft tissue mass without calcification was revealed. CT-scan examination was done and it demonstrated a minimally enhancing low-attenuating soft tissue mass of size 6.3 × 5.6 × 3.0 cm, localized in posterior chest wall in right para-vertebral and right intra-scapular region (Figure 3A & 3B). There was no intra-spinal or intra-thoracic extension. Possibility of RMS or neurofibroma was raised on CT. Fine needle aspiration cytology (FNAC) of mass remained inconclusive. On 5^th^ post-natal-day day, total excision of the mass was done (Figure 4A). Intraoperatively mass could be easily separable from the surroundings. Histopathological examination showed features of embryonal RMS (Figure 4B). Specimen was sent for immuno-histochemical studies which came out to be positive for vimentin, myogenin and muscle specific actin. Patient was referred for chemotherapy which was taken. At 6 months of follow-up he is doing well without recurrence, although 6 months period is too early to comment on recurrence.

## Discussion

Rhabdomyosarcoma (RMS), a malignant tumor of immature mesenchymal cell origin, is the most common soft tissue sarcoma in the pediatric age group, accounting for approximately 3-5% of all childhood malignancies [1]. The median age at diagnosis is five years and almost two thirds of all patients are diagnosed before 10 years of age, the tumor being rare in adults [6]. However, occurrence of RMS in the neonatal period is extremely rare: approximately, only 1-2% of all cases are congenital [6,7]. There are only a few reports about neonatal RMS in the literature [3,4]. Of 3,217 patients registered in the Intergroup Rhabdomyosarcoma Study (IRS) I-IV, only 14 were in the neonatal period at the time of diagnosis [8]. Report from the Italian Cooperative Group by Ferrari et al. [7] among 50 infants with RMS over 20 years, 15 were considered as having congenital RMS. Rodriguez et al. [9], reported only four patients with neonatal RMS treated during 37 years (1962-1999). Thus knowledge about RMS in this age group is scars [3,10].

RMS is traditionally subdivided into embryonal, alveolar and pleomorphic. Pleomorphic RMS, in contrast to embryonal and alveolar RMS, almost exclusively occurs in adults (median age sixth decade). Alveolar RMS represents about 20% of all RMS [11]. Embryonal RMS is the most common type (60-70% of all RMS) [8,10], and is the most predominant in neonates, infants and young children [5,12].

Primary soft-tissue sarcomas of the chest wall are uncommon. RMS usually occurs in the head and neck region (particularly the orbit, nasopharynx, middle ear and oral cavity), bile ducts, retroperitoneum, urogenital tract and extremities [2]. In the first three IRS trials, 35% to 40% of all tumors arose from the head and neck region, about 25% from the genitourinary tract, approximately 20% from extremities, 10% from truncal primary tumors and the remaining 10% from other miscellaneous sites [10]. Thus, chest wall RMS is a relatively rare finding with a reported incidence of 3.7% in the IRS II and IRS III studies [5]. Most of the reports of chest wall involvement are either case reports or small series [5,13] these too in childhood age group.

It has been noted in congenital RMS that the disease may be metastatic at the time of birth, with metastases described in a number of organs and in the placenta too [14].

MRI is the primary imaging modality in RMS with its superior ability to depict soft-tissue changes. CT of the chest is mandatory in order to assess pulmonary metastases from RMS [11]. In our case CT scan of chest and upper abdomen was done, which suggested the possibility of RMS or neurofibroma. Even FNAC did not yield any conclusive result.

Age less than one year has emerged as an independent poor prognostic factor for RMS [8,15]. Regarding the site, RMS occurring at the trunk, para-meningeal areas and the extremities, has been mentioned to be associated with short survival rates. These sites are referred to as non-favourable sites. On the other hand, patients with primary lesions at non-parameningeal regions of the head and neck, and the genitourinary system, had long survival rates [16]. The chest wall is an unfavorable site [17]. Histological features of necrosis and small round cell pattern is liked with a poor prognosis, regardless of final histological diagnosis [8]. Therefore, age of the patient, location of the tumor, histopathologic features and metastatic status all are important prognostic factors for RMS [18].

Treatment of neonatal RMS requires a multidisciplinary approach, where surgery and chemotherapy both have their own specific role. Complete resection of chest wall rhabdomyosarcoma is recommended [19]. Embryonal RMS generally respond very well to chemotherapy [17]. However, a prolonged follow-up is necessary to evaluate the outcome of treatment. Good response to chemotherapy allows surgery to be done less aggressively if needed [20]. The use of radiotherapy is restricted by very high risk of side effects [7] and it should be avoided in the newborns [21]. Thus the role of paediatric oncologists, radiologists, paediatric surgeons and pathologists all is important [12].

In conclusion, we report a rare case of posterior chest wall congenital embryonal rhabdomyosarcoma in a newborn, which responded well to surgery and chemotherapy. In a newborn presenting with a hard mass arising from chest wall, RMS should be considered as one of the differential diagnoses.

## Abbreviations

CT: Computer tomography
FNAC: Fine-needle aspiration cytology
RMS: Rhabdomyosarcoma

## References

[bib-001] Pappo AS, Lyden E, Breneman J, Wiener E, Teot L, Meza J, Crist w, Vietti T (2001). Up-front window trial of topotecan in previously untreated children and adolescents with metastatic rhabdomyosarcoma: an intergroup rhabdomyosarcoma study. J Clin Oncol.

[bib-002] Gordon MS, Hajdu SI, Bains MS, Burt ME (1991). Soft tissue sarcomas of the chest wall: Results of surgical resection. J Thorac Cardiovasc Surg.

[bib-003] Buyukpamukcu M, Varan A, Tanyel C, Senocak ME, Gogus S, Akyuz C, Kutluk T, Buyukpamukcu N (2003). Solid tumors in the neonatal period. Clin Pediatr.

[bib-004] Isaacs H (1987). Congenital and neonatal malignant tumors: a 28-year experience at Children’s Hospital of Los Angeles. Am J Pediatr Hematol Oncol.

[bib-005] Soyer T, Karnak I, Ciftci AO, Senocak ME, Tanyel FC, Büyükpamukçu N (2006). The results of surgical treatment of chest wall tumors in childhood. Pediatr Surg Int.

[bib-006] Perin C, Lacour JP, Thyss A, Michiels JF, Rostain G, Valla J, Ortonne JP (1988). Subcutaneous rhabdomyosarcoma in children; Clinical, Immunologic and ultrastructural aspects. Ann Dermatol Venereol.

[bib-007] Ferrari A, Casanova M, Bisogno G, Zanetti I, Cecchetto G, De Bernardi B, Riccardi R, Tamaro P, Meazza C, Alaggio R, Ninfo V, Carli M (2003). Rhabdomyosarcoma in infants younger than one year old; a report from the Italian Cooperative Group. Cancer.

[bib-008] Lobe TE, Wiener ES, Hays DM, Lawrence WH, Andrassy RJ, Johnston J, Wharam M, Webber B, Ragab A (1994). Neonatal rhabdomyosarcoma: the IRS experience. J Pediatr Surg.

[bib-009] Rodriguez-Galindo C, Hill DA, Onyekwere O, Pin N, Rao BN, Hoffer FA, Kun LE, Pappo AS, Santan VM (2001). Neonatal alveolar rhabdomyosarcoma with skin and brain metastases. Cancer.

[bib-010] Wexler L, Helman L, Williams C, Pizzo PA, Poplack DG (2002). Rhabdomyosarcoma and undifferentiated sarcoma. Principles and Practice of pediatric oncology.

[bib-011] Van Rijn R, Wilde J, Bras J, Merks J (2008). Imaging findings in noncraniofacial childhood rhabdomyosarcoma. Pediatr Radiol.

[bib-012] Parkes SE, Muir KR, Southern L, Cameron AH, Darbyshire PJ, Stevens MC (1994). Neonatal tumours: a thirty year population-based study. Med Pediatr Oncol.

[bib-013] Saenz NC, Ghavimi F, Gerald W, Gollamudi S, LaQuaglia MP (1997). Chest wall rhabdomyosarcoma. Cancer.

[bib-014] White FV, Dehner LP, Belchis DA, Conard K, Davis MM, Stocker T, Zuppan CW, Biegel JA, Perlman EJ (1999). Congenital disseminated malignant rhabdoid tumor: a distinct clinicopathologic entity demonstrating abnormalities of chromosome 22q11. Am J Surg Pathol.

[bib-015] Dillon PW, Whalen TV, Azizkhan RG, Hasse GM, Coran AG, King DR, Smith M (1995). Neonatal soft tissue sarcomas: the influence of pathology on treatment and survival. Children’s Cancer Group Surgical Committee. J Pediatr Surg.

[bib-016] Lawrence W, Anderson JR, Gehan EA, Maurer H (1997). Pretreatment TNM staging of childhood rhabdomyosarcoma: a report of the Intergroup Rhabdomyosarcoma Study Group. Children’s Cancer Study Group. Pediatric Oncology Group. Cancer.

[bib-017] Baker KS, Anderson JR, Link MP, Grier HE, Qualman SJ, Maurer HM, Grier HE, Qualman SJ, Maurer HM, Breneman JC, Wiener ES, Crist WM (2000). Benefit of intensified therapy for patients with local or regional embryonal rhabdomyosarcoma: Results from the Intergroup Rhabdomyosarcoma Study IV. J Clin Oncol.

[bib-018] Güra A, Tezcan G, Karagüzel G, Çevikol C, Oygür N (2007). An unusual localization of embryonal rhabdomyosarcoma in a neonate. Turk J Pediatr.

[bib-019] Saenz NC, Ghavimi F, Gerald W, Gollamudi S, LaQuaglia MP (1997). Chest wall rhabdomyosarcoma. Cancer.

[bib-020] Andrassy R, Spits LE, Coran A (1995). Rhabdomyosarcoma. Pediatric Surgery.

[bib-021] Arcecia R, Weinstein H, MacDonald MG, Mullet MD, Seshia MM (2005). Neoplasia. Avery’s Neonatology, Pathophysiology & Management of the Newborn.

